# Association of *APOE *polymorphism with chronic kidney disease in a nationally representative sample: a Third National Health and Nutrition Examination Survey (NHANES III) Genetic Study

**DOI:** 10.1186/1471-2350-10-108

**Published:** 2009-10-23

**Authors:** Audrey Y Chu, Rulan S Parekh, Brad C Astor, Josef Coresh, Yvette Berthier-Schaad, Michael W Smith, Alan R Shuldiner, Wen Hong L Kao

**Affiliations:** 1 Department of Epidemiology, Bloomberg School of Public Health, Johns Hopkins University, Baltimore, MD, USA; 2 Hospital for Sick Children, Sick Kids Research Institute, University Health Network and University of Toronto, Toronto, Ontario, Canada; 3 Laboratory of Genomic Diversity, National Cancer Institute, Frederick, MD, USA; 4 Basic Research Program, SAIC-Frederick, National Cancer Institute, Frederick, MD, USA; 5 Division of Endocrinology, Diabetes and Nutrition, University of Maryland School of Medicine, Baltimore, MD, USA

## Abstract

**Background:**

Apolipoprotein E polymorphisms (*APOE*) have been associated with lowered glomerular filtration rate (GFR) and chronic kidney disease (CKD) with e2 allele conferring risk and e4 providing protection. However, few data are available in non-European ethnic groups or in a population-based cohort.

**Methods:**

The authors analyzed 5,583 individuals from the Third National Health and Nutrition Examination Survey (NHANES III) to determine association with estimated GFR by the Modification of Diet in Renal Disease (MDRD) equation and low-GFR cases. Low-GFR cases were defined as GFR <75 ml/min/1.73 m^2^; additionally, GFR was analyzed continuously.

**Results:**

In univariate analysis, the e4 allele was negatively associated with low-GFR cases in non-Hispanic whites, odds ratio (OR): 0.76, 95% confidence interval (CI): 0.60, 0.97. In whites, there was a significant association between increasing *APOE *score (indicating greater number of e2 alleles) and higher prevalence of low-GFR cases (OR: 1.21, 95%CI: 1.01, 1.45). Analysis of continuous GFR in whites found the e4 allele was associated with higher levels of continuous GFR (β-coefficient: 2.57 ml/min/1.73 m^2^, 95%CI: 0.005, 5.14); in non-Hispanic blacks the e2 allele was associated with lower levels of continuous GFR (β-coefficient: -3.73 ml/min/1.73 m^2^, 95%CI: -6.61, -0.84). *APOE *e2 and e4 alleles were rare and not associated with low-GFR cases or continuous GFR in Mexican Americans.

**Conclusion:**

In conclusion, the authors observed a weak association between the *APOE *e4 allele and low-GFR cases and continuous GFR in non-Hispanic whites, and the *APOE *e2 allele and continuous GFR in non-Hispanic blacks, but found no association with either measure of kidney function in Mexican Americans. Larger studies including multiethnic groups are needed to determine the significance of this association.

## Background

Chronic kidney disease (CKD) is a major public health issue in the United States; CKD prevalence is estimated to be between 4.8% and 7.0% in U.S. adults, with higher prevalence observed in US non-Hispanic whites (7.5%) and non-Hispanic blacks (7.8%) and lower prevalence in Mexican Americans (1.8%) [1]. The discrepancy in prevalence of disease by ethnicity may be due to differences in access to healthcare, prevalence of modifiable lifestyle risk factors for CKD, or in genetic risk factors [2,3]. Kidney function can be estimated by the continuous glomerular filtration rate (GFR) using various equations, including the Modification of Diet in Renal Disease (MDRD) equation [4,5]. Individuals with low GFR are considered to have CKD, which is commonly defined by a GFR <60 ml/min/1.73 m^2 ^using the MDRD equation [1,6].

Both continuous GFR and CKD are heritable [7,8] and previous studies, including our own work, suggest *APOE *genetic variations may contribute to risk of CKD and low GFR. *APOE *polymorphisms have been the focus of several studies investigating lipid transport in the kidney [9-23]. The ability of ApoE to bind and clear lipids in the kidney is directly related to structural instability and repair of the glomerular lining of the kidney [11,24,25]. ApoE glycoprotein forms are coded by three *APOE *alleles, e2, e3 and e4 [26,27]. The e3 form is the most common and is not associated with increased risk of CKD. e4 has been associated with higher GFR and decreased risk of CKD [28] but increases risk of Alzheimer's disease [29,30] and coronary artery disease [31]. On the other hand, e2, the rarest ApoE isoform [28,32] has been shown to be associated with increased risk of CKD and lower GFR although it affords protection against Alzheimer's disease [33,34] and lowers cholesterol levels [35].

We previously demonstrated an association between *APOE *polymorphisms and incident CKD in the Atherosclerosis Risk in Communities (ARIC) study, a large community based prospective study of middle-aged white and African-American adults [28]. In the present study, we further investigate the association between *APOE *polymorphisms and low-GFR cases in a large nationally representative population-based sample of non-Hispanic Whites, non-Hispanic blacks and Mexican Americans from the Third National Health and Nutrition Examination Survey (NHANES III). The goals of this study are to 1) obtain population-based estimates of *APOE *allele and genotype frequencies across a wide range of age groups and by three ethnicities in the US; 2) determine the associations between *APOE *polymorphisms and low-GFR cases by ethnicity; and 3) determine whether allele frequencies can account for part of the differences in prevalence of low-GFR cases between populations.

## Methods

### Study population

Data from a subset of NHANES III participants who consented to genetic research and were successfully genotyped were used in the present analysis (n = 7,159). The NHANES III examinations were carried out from 1988 to 1994 by the National Center for Health Statistics using a complex multistage probability sampling design [36,37]. Sample weights were applied to the population to correct for non-response and unequal probability of selection.

DNA was obtained by growing cell lines from blood samples collected from consenting participants over the age of 12 during the second phase of NHANES III from 1991 to 1994. To avoid non-response bias, original sample weights were recalculated for the genetic subset [38,39].

Informed consent has been obtained from patients when appropriate. Procedures were followed in accordance with ethical standards of the Johns Hopkins School of Public Health Office of Human Subjects Research and Institutional Review Board.

### Exclusions

The following exclusion criteria were applied to derive the final analytical sample size of 5,583: missing genotypes (n = 80), self-reported "other" ethnicity due to small sample size (n = 333), missing serum creatinine measurement (n = 31), or younger than 20 years (n = 1,119). After exclusion, the total number of subjects available for analysis was 5,583 (2,328 non-Hispanic whites, 1,599 non-Hispanic blacks and 1,656 Mexican Americans).

### Outcome - Glomerular Filtration Rate

Serum creatinine (*SCr*) measures using modified kinetic Jaffe reaction were calibrated to standardized creatinine reference methods by multiplying by 0.960 and then subtracting 0.184 mg/dl from the recorded values [40]. The MDRD equation was used to estimate GFR [41]:



In addition to the MDRD equation, the newly developed CKD-EPI formula [see Additional file 1] was also used to estimate GFR[42]. GFR values >200 ml/min/1.73 m^2 ^were set to have the maximum value of 200 ml/min/1.73 m^2 ^[1].

Given the low count of CKD cases (as defined by GFR <60 ml/min/1.73 m^2^) in the smaller NHANES III Genetics Study and the low frequency of the *APOE *e2 allele, we defined low-GFR cases as individuals having GFR <75 ml/min/1.73 m^2 ^and controls having GFR ≥ 75 ml/min/1.73 m^2 ^[43].

### *APOE *genotyping

The *APOE *polymorphism consists of two single nucleotide polymorphisms (SNPs), *APOE *Cys112Arg (rs429358; T to C nucleotide substitution) and *APOE *Arg158Cys (rs7412; C to T nucleotide substitution). The native *APOE *e3 allele consists of Cys112 and Arg158; the e2 allele is a combination of Cys112 and 158Cys; and the e4 allele is a combination of 112Arg and Arg158. Genotyping of the two SNPs was performed using the TaqMan assay as previously described [28]. Percent agreement for 440 replicates was 100%. Both SNPs were in Hardy-Weinberg proportion.

### Covariates

Demographic and health status information were collected during detailed home interviews and extensive physical exams at Mobile Examination Centers (MEC) or in home examinations and have been described in detail [36]. Age, sex and ethnicity were self reported, with ethnicity selected from non-Hispanic white, non-Hispanic black, Mexican-American or other. Variables included in analysis obtained during the interview include: smoking (ever/never smoked) and educational status (less than high school degree/at least high school degree). Body mass index (BMI) was defined by height and weight measured at the exam. Blood samples were collected by venipuncture for measurement of serum creatinine (SCr), blood glucose, total serum cholesterol, high density lipoprotein (HDL) cholesterol, thyroid stimulating hormone (TSH), triglycerides and C-reactive protein (CRP) according to NHANES III lab protocols [44,45].

Diabetes status was established by self-report of diabetes, self-report of diabetes medication, fasting plasma glucose ≥ 7.0 mmol/l (≥126 mg/dl), or a 2-hr glucose level ≥ 11.1 mmol/l (≥200 mg/dl) after a 75-g oral glucose tolerance test. Systolic and diastolic blood pressures were determined by the average of 3 blood pressure measurements taken during the examination. Participants were classified as hypertensive by self-report of hypertension, report of anti-hypertensive medication, systolic blood pressure ≥ 140 mmHg or diastolic blood pressure ≥ 90 mmHg. High blood cholesterol was defined by self-reported high blood cholesterol, report of lipid-lowering medications or total serum cholesterol ≥ 6.2 mmol/l (≥ 240 mg/dl). Obesity was defined by BMI ≥ 30 kg/m^2^. Prevalent cardiovascular disease was ascertained by self-report of coronary heart disease, myocardial infarction, or stroke. Urine albumin was measured using a solid-phase fluorescent immunoassay and urine creatinine was measured using the Jaffe rate reaction [45]; the urinary albumin-creatinine ratio (ACR) was calculated as ug/mg. Albuminuria was defined as ACR>30 ug/mg by the National Kidney Foundation.

### Statistical analysis

Analyses were weighted to account for the sampling design of NHANES III; recalculated weights from NHANES III were applied to NHANES III Genetic Study. The "survey" command in Stata 10.0 was used for all analyses [46]. Analyses were stratified by self-reported ethnicity. Allele frequencies and genotype distributions were calculated with and without sampling weights; the two sets of results had minimal differences.

All alleles were modeled additively. Prior research indicated the e2 allele as the at-risk allele for low-GFR/CKD, e4 as the protective allele, and e3 as the reference. A summary score was used to model *APOE *variation by designating each e2, e3 and e4 allele +1, 0 and -1 points, respectively [28]. Using this model, +2 points were assigned to individuals of genotype e2/e2, +1 point for e2/e3, 0 points for e3/e3 and e2/e4, -1 points for e3/e4 and -2 points for e4/e4 individuals [28].

The association between low-GFR cases and *APOE *polymorphism was assessed using logistic regression. Models estimating allele-specific odds ratios are adjusted for the e2 and the e4 allele simultaneously. The minimally adjusted model includes age and sex. Covariate selection was based on *a priori *hypotheses of risk factors and comorbidities; the final fully adjusted model included age, sex, education, ever/never smoking, CRP, triglycerides, HDL cholesterol, TSH, diabetes, hypertension and obesity. In addition, we wanted to explore the pathway between *APOE *and low-GFR independent of albuminuria (ACR > 30 ug/mg, as defined by the National Kidney Foundation), representing evidence of physical kidney damage. We performed an analysis using the univariate model adjusted for presence of albuminuria and added presence or absence of albuminuria to our final model. Analysis of the association between *APOE *polymorphisms and continuous GFR was assessed using linear regression.

Power calculations were carried out using Quanto [47] and POWER V3.0 [48].

## Results

### Population characteristics

A summary of characteristics, by ethnicity and case-control status, of the 5,583 NHANES III participants included in the present study are presented in table 1. Non-Hispanic white participants had a mean age of 46.2 years, had the lowest BMI and were more likely to have dyslipidemia but less likely to have diabetes. The mean age of the non-Hispanic black participants was 41.8 years, and they had the highest prevalence of diabetes, hypertension, cardiovascular disease, and obesity. The mean age of the Mexican Americans was only 37.4 years, which probably accounted for the observation that they tended to have lower prevalence of existing cardiovascular risk factors shown in table 1.

**Table 1:** Estimated weighted population characteristics of 5,583 NHANES III Genetic Study (1991-1994) participants by ethnicity*

	**Non-Hispanic white**		**Non-Hispanic black**		**Mexican American**	
	
	***Mean/%***	***SE***	***Mean/%***	***SE***	***Mean/%***	***SE***
**Number of subjects **(n)	2,328		1,599		1,656	
**Age **(y)	46.2	0.9	41.8	0.8	37.4	0.6
**Body mass index **(kg/m^2^)	26.6	0.2	28.2	0.3	27.8	0.9
**Male **(%)	48.1	1.0	44.2	1.3	52.1	1.0
**Diabetes **(%)	6.8	0.6	9.4	0.8	8.4	0.9
**Hypertension **(%)	29.4	1.9	38.2	1.7	22.7	1.4
**High blood cholesterol **(%)	30.4	1.0	20.8	0.9	16.6	1.0
**Cardiovascular disease **(%)	8.9	0.9	10.8	0.8	7.5	0.7
**Obesity **(%)	23.1	1.2	32.8	1.2	29.0	0.8
**High school degree **(%)	82.9	1.2	68.3	2.4	55.5	2.7
**Serum creatinine **(mg/dL)	0.86	0.01	0.93	0.01	0.78	0.00
**Never smoked **(%)	74.3	1.9	68.6	1.0	78.6	1.2
**Cystatin C **(mg/dL)	0.98	0.02	0.94	0.03	0.87	0.02
**Albumin to creatinine ratio **	18.88	1.74	37.06	4.90	53.15	14.61
**Triglycerides **(mg/dL)	148.98	2.94	117.71	1.74	161.63	4.03
**High density lipoprotein cholesterol **(mg/dL)	49.73	0.63	54.53	0.54	47.49	0.50
**C-reactive protein **(mg/L)	0.40	0.02	0.52	0.02	0.46	0.03
**Estimated glomerular filtration rate **(ml/min/1.73 m^2^)	88.2	0.8	101.4	0.9	103.7	0.7
**Low-glomerular filtration rate cases **(GFR <75) (%)	22.8	1.5	11.2	1.2	6.4	0.8

* All between ethnicity group comparisons are statistically significant, p < 0.05.

Prevalence of low-GFR (<75 ml/min/1.73 m^2^) by the MDRD equation was the lowest in Mexican Americans (6.4%, estimated from 174 cases) compared to either non-Hispanic whites (22.8%, estimated from 792 cases) or non-Hispanic blacks (11.2%, estimated from 186 cases).

### Allele and genotype frequency

The e2 allele was the rarest allele for each ethnicity, with population frequencies estimated at 3.4%, 9.7% and 7.8% in Mexican Americans, non-Hispanic blacks and non-Hispanic whites, respectively (table 2). Frequency of the e4 allele was 10.8% among Mexican Americans, compared to 15% in whites and 22% in non-Hispanic blacks. The most common genotypes e2/e3, e3/e3, and e3/e4 made up 95% of the non-Hispanic white genotypes, 89% of non-Hispanic black genotypes and 97% of Mexican-American genotypes. The e2/e2 genotype was rare in all groups (1% in non-Hispanic whites and blacks and 0.1% in Mexican Americans) and was found in only one Mexican American participant.

**Table 2:** *APOE *allele and genotype counts and population frequencies* of NHANES III Genetic Study (1991-1994) by ethnicity

	**Non-Hispanic white**		**Non-Hispanic black**		**Mexican American**	
	
***Allele/Genotype***	***count***	***population frequency***	***count***	***population frequency***	***count***	***Population frequency***
e2	407	0.08	326	0.10	115	0.03
e3	3,557	0.77	2,186	0.68	2,854	0.86
e4	692	0.15	686	0.22	343	0.11
						
e2/e2	17	0.01	9	0.01	1	0.001
e2/e3	315	0.13	237	0.14	100	0.06
e2/e4	58	0.02	71	0.05	13	0.01
e3/e3	1,354	0.58	745	0.46	1,238	0.74
e3/e4	534	0.24	459	0.29	278	0.17
e4/e4	50	0.02	78	0.05	26	0.02

* The count is based on samples in NHANES III; the population frequency is estimated from the number of samples using sampling weights from NHANES III

### Univariate association between *APOE *variants with low-GFR cases by MDRD equation in case-control analysis

Each e4 allele was inversely associated with low-GFR cases in non-Hispanic whites (OR: 0.76, 95%CI: 0.60, 0.97) as shown in table 3. In non-Hispanic blacks, e4 was seen with slightly lower odds of low-GFR (OR: 0.95, 95%CI: 0.69, 1.32), but the association was not significant. There was no association of the e2 allele and low-GFR in any of the three ethnicities. Associations between *APOE *variations and low-GFR in Mexican Americans were not consistent with the findings among whites and blacks in univariate or multivariate analyses.

**Table 3:** Odds Ratios of low-GFR cases (GFR < 75 ml/min/1.73 m2) by the MDRD equation and CKD-EPI formula associated with the e2 allele, e4 allele, and *APOE *score in NHANES III Genetic Study (1991-1994) participants by ethnicity

	***APOE *e2**		***APOE *e4**		***APOE *score**	
	
	***OR***	***95%CI***	***OR***	***95%CI***	***OR***	***95%CI***
**Non-Hispanic white**						
*MDRD equation*						
Crude	1.08	0.81, 1.45	0.76*	0.60, 0.97	1.21*	1.01, 1.45
Minimally adjusted	0.96	0.66, 1.39	0.82	0.60, 1.14	1.10	0.88, 1.38
Final	0.99	0.69, 1.44	0.83	0.60, 1.15	1.11	0.87, 1.42
*CKD-EPI formula*						
Crude	1.21	0.82, 1.79	0.72*	0.59, 0.88	1.31*	1.06, 1.60
Minimally adjusted	1.10	0.61, 1.99	0.82	0.65, 1.05	1.16	0.90, 1.50
Final	1.12	0.59, 2.14	0.77	0.58, 1.02	1.22	0.92, 1.62
**Non-Hispanic black**						
*MDRD equation*						
Crude	1.45	0.91, 2.33	0.95	0.69, 1.32	1.19	0.96, 1.49
Minimally adjusted	1.50	0.91, 2.50	1.01	0.72, 1.42	1.16	0.92, 1.46
Final	1.37	0.79, 2.38	1.07	0.75, 1.53	1.08	0.87, 1.36
*CKD-EPI formula*						
Crude	1.29	0.69, 2.41	0.88	0.67, 1.18	1.19	0.89, 1.59
Minimally adjusted	1.42	0.73, 2.76	0.92	0.70, 1.21	1.20	0.86, 1.68
Final	1.26	0.63, 2.52	0.94	0.68, 1.29	1.13	0.83, 1.57
**Mexican American**						
*MDRD equation*						
Crude	0.71	0.34, 1.48	1.12	0.69, 1.79	0.85	0.57, 1.28
Minimally adjusted	0.77	0.34, 1.72	1.13	0.71, 1.82	0.85	0.57, 1.28
Final	0.77	0.34, 1.81	1.14	0.71, 1.86	0.85	0.57, 1.27
*CKD-EPI formula*						
Crude	0.90	0.45, 1.80	0.90	0.48, 1.66	1.05	0.66, 1.67
Minimally adjusted	0.97	0.44, 2.12	0.92	0.43, 1.99	1.05	0.62, 1.79
Final	0.95	0.37, 2.42	0.85	0.39, 1.85	1.10	0.66, 1.84

Crude model adjusted for e2 and e4 allele

Minimally adjusted model includes the crude model plus age and sex

Final model includes the minimally adjusted model plus education, ever/never smoking, CRP, triglycerides, HDL cholesterol, TSH, obesity, diabetes, hypertension and presence of albuminuria

*p < 0.05

Similar results were obtained in univariate *APOE *summary score analysis and prevalence of low-GFR (table 3). In non-Hispanic whites, each increasing *APOE *summary score point (representing greater number of e2 alleles) significantly increased the odds of low-GFR by 21% (OR: 1.21, 95%CI: 1.01, 1.45), therefore individuals with the e2/e2 genotype have a 21% increased odds of low-GFR compared to those with the e2/e3 genotype. We observed a similar trend of increasing summary score with low-GFR cases in non-Hispanic blacks, but the association was not significant (table 3). No consistent association of *APOE *score and low-GFR cases was seen among Mexican Americans.

Power calculations were performed for association between the e4 allele, the e2 allele, and *APOE *score with low-GFR cases by ethnicity. α was set at 0.05 for all calculations. In non-Hispanic whites, with a fixed sample size of 2,328 and observed prevalence of low-GFR cases of 22.8%, and an e4 allele frequency of 0.15, we have 80% power to detect an odds ratio of 0.76. Using the same sample size and disease prevalence parameters, with an e2 allele frequency of 0.08, we have 31% power to detect an odds ratio of 1.20. Using the same sample size and disease prevalence parameters, with the observed *APOE *score distribution for non-Hispanic whites from (table 2), we have 71% power to detect an odds ratio of 1.20.

In non-Hispanic blacks, with a fixed sample size of 1,599, observed prevalence of low-GFR cases of 11.2%, and an e4 allele frequency of 0.22, we have 53% power to detect an odds ratio of 0.76. Using the same sample size and disease prevalence parameters, with an e2 allele frequency of 0.11, we have 17% power to detect an odds ratio of 1.20. Using the same sample size and disease prevalence parameters, with the observed *APOE *score distribution for non-Hispanic blacks from (table 2), we have 44% power to detect an odds ratio of 1.20.

In Mexican Americans, with a fixed sample size of 1,656, observed prevalence of low-GFR cases of 6.4%, and an e4 allele frequency of 0.11, we have 22% power to detect an odds ratio of 0.76. Using the same sample size and disease prevalence parameters, with an e2 allele frequency of 0.03, we have 7% power to detect an odds ratio of 1.20. Using the same sample size and disease prevalence parameters, with the observed *APOE *score distribution for Mexican Americans from (table 2), we have 18% power to detect an odds ratio of 1.20.

### Multivariate association between *APOE *variants with low-GFR cases by MDRD equation in case-control analysis

In non-Hispanic whites, adjusting for age and sex revealed a suggestive association between the e4 allele and decreased prevalence of low-GFR in non-Hispanic whites (table 3). This adjustment, however, attenuated the association of the e4 allele and low-GFR and diminished significance. The final multivariate models included *APOE *variants, age, sex, education, ever/never smoking, HDL cholesterol, CRP, triglycerides, TSH, diabetes, hypertension, obesity and albuminuria. In our final model, the *APOE *e4 allele tended to associate with lower prevalence of low-GFR cases in Non-Hispanic whites. No significant associations were observed with the e2 allele, although the odds ratios were consistently greater than the null in non-Hispanic blacks.

Although score analysis of our final multivariate model supported findings from the additive genetic models, i.e. per point increase in *APOE *summary score tended to associated with low-GFR cases in non-Hispanic whites (OR: 1.11, 95%CI: 0.87, 1.42) and blacks (OR: 1.08, 95%CI: 0.87, 1.36), these estimates did not reach statistical significance.

### Association between *APOE *variants with low-GFR cases by MDRD equation in case-control analysis adjusting for albuminuria

To examine the association of *APOE *alleles and low-GFR cases independent of kidney damage, we adjusted for albuminuria (ACR >30 ug/mg) in our univariate models. Table 4 shows models adjusted for albuminuria by ethnicity. Adjustment for albuminuria in the non-Hispanic whites showed that *APOE *e4 allele tended to associate with low-GFR cases (OR: 0.77, 95%CI: 0.59, 1.01, p = 0.06). Moreover, the *APOE *score summary was also significantly associated with low-GFR cases after adjusting for albuminuria (OR: 1.21, 95%CI: 1.00, 1.46). Consistent positive associations of *APOE *score summary and low-GFR cases were seen in non-Hispanic blacks after adjustment for albuminuria. However, none of the associations attained statistical significance (table 4).

**Table 4:** Odds Ratios of GFR < 75 ml/min/1.73 m^2 ^by the MDRD equation associated with the e2 allele, the e4 allele, and *APOE *score adjusted for albuminuria in NHANES III Genetic Study (1991-1994) participants by ethnicity

	***APOE *e2**		***APOE *e4**		***APOE *score**	
	
	***OR***	***95%CI***	***OR***	***95%CI***	***OR***	***95%CI***
**Non-Hispanic whites**	1.10	0.81, 1.48	0.77	0.59, 1.01	1.21*	1.00, 1.46
**Non-Hispanic blacks**	1.34	0.80, 2.25	0.90	0.65, 1.24	1.19	0.995, 1.44
**Mexican Americans**	0.77	0.36, 1.68	1.14	0.72, 1.80	0.85	0.56, 1.31

Models adjusted for the e2 and e4 allele, and albuminuria (albumin-creatinine ratio >30 ug/mg)

*p < 0.05

### Association of *APOE *and continuous GFR by MDRD equation

In non-Hispanic whites, analysis of continuous GFR estimated by the MDRD equation, each e4 allele was associated with a 2.57 ml/min/1.73 m^2 ^higher GFR level (95%CI: 0.005, 5.14 ml/min/1.73 m^2^). The e2 allele was not associated with GFR level (β: 1.23 ml/min/1.73 m^2^, 95%CI: -1.90, 4.38) in whites. In non-Hispanic blacks, the e2 allele associated with a 3.73 ml/min/1.73 m^2 ^lower GFR level (95%CI: -6.61, -0.84 ml/min/1.73 m^2^). The e4 allele tended towards association with lower GFR level in blacks (β: -1.63 ml/min/1.73 m^2^, 95%CI: -4.38, 0.18), but was not significant.

### Association of *APOE *and low GFR by CKD-EPI Equation and continuous GFR by CKD-EPI equation

To confirm that the association between *APOE *and low GFR was not specific to the MDRD equation, we also estimated GFR using the newly developed CKD-EPI equation [49]. The CKD-EPI formula estimated lower prevalence of low-GFR cases compared to the MDRD formula (4.1% in non-Hispanic whites, 16.0% in non-Hispanic blacks and 11.2% in Mexican Americans). Despite different prevalence estimates, similar associations were observed in analysis of *APOE *polymorphisms and *APOE *score method with low-GFR cases in non-Hispanic whites and blacks in univariate and multivariate analyses (Table 3). Continuous analysis of GFR by CKD-EPI equation revealed similar associations as continuous analyses of GFR by MDRD equation in both non-Hispanic whites and non-Hispanic blacks (data not shown).

## Discussion

Following on previous work, we replicated the inverse association between *APOE *e4 allele and low-GFR cases in non-Hispanic whites. The *APOE *e4 allele was significantly associated with decreased prevalence of low-GFR cases in non-Hispanic whites, and higher levels of continuous GFR. Individuals with a copy of the e4 allele had 24% lower odds of a GFR less than 75 ml/min/1.73 m^2^, and, on average, a 2.57 ml/min/1.73 m^2 ^higher GFR. The e2 allele did not associate with low-GFR cases (or lower levels of continuous GFR). We observed a weak, but significant, association between increasing *APOE *summary score points (greater number of e2 alleles) and higher prevalence of low-GFR cases. Additionally, this observed association between *APOE *summary score and low-GFR cases was independent of a kidney damage mechanism, as seen in the model adjusting for albuminuria. We had previously implemented the *APOE *summary score to detect an association of *APOE *variation with kidney function [28], and obtained similar effects after full adjustment for confounders. In light of previously published studies demonstrating positive relationship between *APOE *variants and CKD, the suggestive evidence from this study, and considering the wide age range and the non-clinical nature of NHANES III, we believe our results support and replicate the association between genetic variations of *APOE *and low-GFR cases in non-Hispanic whites.

While we did not observe a significant association of *APOE *polymorphisms with prevalence of low-GFR in non-Hispanic blacks due to low power (see **Univariate Association **under **Results**) we did observe an association between the e2 polymorphism and lower levels of continuous GFR. Additionally, the odds ratios for blacks for e2, e4 and the *APOE *score method from our study are consistent with the hazard ratios of *APOE *and progression of CKD estimated from African Americans in the Atherosclerosis Risk in Communities (ARIC) study [28]. No association was detected between *APOE *polymorphism and prevalence of low-GFR or continuously measured GFR in Mexican Americans. The *APOE *e2 and e4 allele frequencies were very low in Mexican Americans and our analysis did not have enough power to detect an association (see **Univariate Association **under **Results**), even if one existed.

This is the first study of *APOE *and estimated kidney function in Mexican Americans and establishes population-based allele frequencies in a nationally representative population of Mexican Americans. *APOE *variation is uncommon in Mexican Americans. The frequency of the e2 allele is 3.5% and the e4 allele is 11% in Mexican Americans from NHANES III. These estimates are consistent with results from smaller regional cohorts, e2: 2.4 - 4.8% and e4: 6.9 - 9.9% [50-52]. The combination of low allele frequency and low prevalence of estimated GFR <75 ml/min/1.73 m^2 ^(6.4%) in this population resulted in an underpowered analysis to detect the significant association observed in non-Hispanic whites. Moreover, the lack of observed association in this population could be due to the need for a validated equation to estimate kidney function in Mexican Americans, but could also indicate *APOE *variants are not associated with or have minimal effect on kidney function in this population.

The odds ratios obtained from analysis of *APOE *summary score and low-GFR cases were similar to the estimates previously observed in ARIC - an increase in the number of e2 alleles was observed with an increased prevalence of low-GFR cases, or alternately, the decrease in the number of e2 alleles was associated with lower prevalence of low-GFR cases [28]. Our adjusted estimates were not significant, possibly due to the smaller sample size of NHANES III genetic study compared to ARIC. Nevertheless, the direction of association and magnitude of the association are consistent between the two studies.

The inverse association between the e4 allele and prevalence of low-GFR cases (GFR<75 ml/min/1.73 m^2^) is an important finding in determining whether *APOE *variation is a risk factor for kidney disease. Studies of end-stage renal disease (ESRD) patients show lower prevalence of the e4 allele in ESRD than the general population [53]. The e4 allele and e3/e4 - e4/e4 genotypes are found in lower frequency in patients with glomerular nephropathy [21]. The e4 allele was found to be protective for onset and progression of diabetic nephropathy in type 2 diabetes [20,54].

Greater prevalence of the e2 allele has been observed in ESRD patients compared to the general population [53]. The e2 allele was found to be a risk factor for onset and progression of diabetic nephropathy in type 2 diabetes [13]. The e2 allele was also found to be associated with development of diabetic nephropathy in type 1 diabetics [14]. Several studies have produced null results from investigations of ApoE and renal disease, but were generally underpowered [20,55]. A substantial body of research has consistently demonstrated the e2 allele is associated with increased risk of renal dysfunction and the e4 allele is inversely associated with risk of renal disease.

In order to assess whether this relationship is causal, additional investigations into the molecular pathways of different forms of ApoE on the pathophysiology of CKD and kidney function need to be carried out. The pathway by which ApoE acts on the kidney has yet to be precisely delineated. However, dysfunctional kidney repair functions (ineffective remodeling and mesangial cell proliferation) have been implicated in previous studies [24]. Change in membrane permeability also may lead to derangement of kidney function.

Differences in *APOE *genotype frequencies may account for some of the differences in low-GFR prevalence across ethnic subpopulations. In non-Hispanic blacks, we expected higher e2 allele frequencies due to an increased prevalence of CKD, and we expected lower frequencies of the e2 allele in Mexican-Americans due to lower prevalence of CKD in this population. We have identified a very low prevalence of the e2 allele and thus low frequencies of the e2/e2, e2/e3 and e2/e4 genotypes in a population based sample of Mexican-Americans, consistent with previously published results [50,51]. We cannot make further inferences of the effect of e2 on estimated kidney function beyond the consistent, yet non-significant association with higher prevalence of low-GFR.

We chose to use GFR<75 ml/min/1.73 m^2 ^to define cases and not the National Kidney Foundation's (NKF) standard definition for stage 3 CKD (GFR<60 ml/min/1.73 m^2^) since using the NKF definition resulted in severely underpowered analyses (see **Univariate association **under **Results**). Comparison of results from univariate analyses using a case definition of GFR<60 ml/min/1.73 m^2 ^showed similar association of case status with *APOE2 *(OR: 1.07, 95%CI: 0.61, 1.87), *APOE4 *(OR: 0.82, 95%CI: 0.58-1.17) and *APOE *score (OR: 1.15, 95%CI: 0.84-1.56) as our current results with case definition of GFR<75 ml/min/1.73 m^2^, but were not statistically significant. Additionally, similar risk factors have been associated with both cases defined by GFR<75 ml/min/1.73 m^2 ^and cases defined by GFR<60 ml/min/1.73 m^2 ^[43].

This study is large and representative of the general U.S. population, and findings in this study are consistent with previous results. However there are several limitations. First, CKD is clinically defined by altered kidney function or structure for a period of three months or more. The single serum creatinine measurement used in our study to estimate GFR may have lead to misclassification of disease status and decreased power. Second, CKD was defined based on estimated GFR from the MDRD equation and the CKD-EPI formula rather than the gold standard methods of direct measurement of glomerular function using inulin clearance, iothalamate clearance and creatinine clearance. Third, serum creatinine measures are higher in individuals with large amounts of muscle mass [56], in addition to individuals with impaired kidney function. Therefore, the low GFR estimates generated for those with high muscle mass could indicate worse kidney function than in reality. This misclassification could lead to an attenuation of the association between *APOE *and CKD. Fourth, neither the MDRD equation nor the CKD-EPI formula has been validated in Mexican Americans. Lastly, there is the possibility the relationship between *APOE *variation and CKD is due to survivor bias; we observed a slight decline in e4 allele frequency with increasing age in all three ethnic groups. However, analyses stratified by age groups revealed similar associations to unstratified analysis (data not shown).

One of the main strengths of the present study was the large, nationally representative, population-based sample of non-Hispanic whites, non-Hispanic blacks and Mexican Americans. Previous epidemiologic studies have shown risk of CKD associated with *APOE *variants mostly in clinic-based and international samples [13,14,19-21,28,32,53,54]. Our generalizable results provide evidence to support the effects of *APOE *variants on CKD. In addition, reverse causality - often a problem with cross-sectional studies - is not an issue here since *APOE *gene variants should precede all manifestations of CKD, and therefore cannot be affected by the presence of CKD.

## Conclusion

Observational epidemiology studies have supplied population-based evidence of the effect of *APOE *variations on prevalence and progression of kidney disease [28,32]. The e4 allele is consistently associated with decreased risk of CKD/low-GFR in whites. But, we cannot rule out a survival bias as a source of confounding for the association between the e4 allele and higher estimated kidney function. Replication of these findings in a large population of whites is necessary, as are future efforts in large multiethnic populations to understand the possible impact of *APOE *polymorphism in the population. In addition, research of CKD and ApoE should be directed towards the elucidation of the molecular biology of kidney disease and mechanisms through which different forms of *APOE *may play a modulating role. These mechanisms would ideally include the effect of maintenance and repair of kidney structures on the permeability of the glomerular membrane. Additionally, physiology studies would aim to determine what role age and survival play in the effect of *APOE *on kidney unction, as well as validating equations of estimated kidney function in ethnic minorities.

## Abbreviations

ACR: Albumin creatinine ratio; APOE: Apolipoprotein E; ARIC: Atherosclerosis Risk in Communities; BMI: Body mass index; CI: Confidence interval; CKD: Chronic kidney disease; CRP: C-reactive protein; DN: Diabetic nephropathy; eGFR: Estimated glomerular filtration rate; ESRD: End-stage renal disease; GFR: Glomerular filtration rate; HDL: High density lipoprotein cholesterol; MDRD: Modification of Diet in Renal Disease; MEC: Mobile examination center; NHANES III: National Health and Nutrition Examination Survey III; OR: Odds ratio; SNP: Single nucleotide polymorphism; SCr: Serum creatinine; TSH: Thyroid stimulating hormone.

## Competing interests

The authors declare that they have no competing interests.

## Authors' contributions

AYC performed statistical analysis and drafted the manuscript. WHK conceived of the study and participated in its design and coordination. RSP, BCA, JC, YB, MWS, ARS and WHK provided critical revisions of the manuscript. All authors read and approved the final manuscript.

## Pre-publication history

The pre-publication history for this paper can be accessed here:



## Supplementary Material

Additional file 1 **Estimation of GFR using the CKD-EPI formula**. The CKD-EPI formulas are provided. These formulas were used to estimate GFR in our analyses in addition to the MDRD equation. Click here for file

## References

[B1] Coresh J, Astor BC, Greene T, Eknoyan G, Levey AS (2003). Prevalence of chronic kidney disease and decreased kidney function in the adult US population: Third National Health and Nutrition Examination Survey. Am J Kidney Dis.

[B2] Satko SG, Freedman BI (2004). The importance of family history on the development of renal disease. Curr Opin Nephrol Hypertens.

[B3] Freedman BI, Satko SG (2000). Genes and renal disease. Curr Opin Nephrol Hypertens.

[B4] Levey AS, Bosch JP, Lewis JB, Greene T, Rogers N, Roth D (1999). A more accurate method to estimate glomerular filtration rate from serum creatinine: a new prediction equation. Modification of Diet in Renal Disease Study Group. Ann Intern Med.

[B5] Cockcroft DW, Gault MH (1976). Prediction of creatinine clearance from serum creatinine. Nephron.

[B6] Levey AS, Coresh J, Balk E (2003). National Kidney Foundation practice guidelines for chronic kidney disease: evaluation, classification, and stratification. Ann Intern Med.

[B7] Bochud M, Elston RC, Maillard M (2005). Heritability of renal function in hypertensive families of African descent in the Seychelles (Indian Ocean). Kidney Int.

[B8] Fox CS, Yang Q, Cupples LA (2004). Genomewide linkage analysis to serum creatinine, GFR, and creatinine clearance in a community-based population: the Framingham Heart Study. J Am Soc Nephrol.

[B9] Attman PO, Samuelsson O, Alaupovic P (1993). Lipoprotein metabolism and renal failure. Am J Kidney Dis.

[B10] Saito T (1997). Abnormal lipid metabolism and renal disorders. Tohoku J Exp Med.

[B11] Mahley RW, Rall SC (2000). Apolipoprotein E: far more than a lipid transport protein. Annu Rev Genomics Hum Genet.

[B12] Blue ML, Williams DL, Zucker S, Khan SA, Blum CB (1983). Apolipoprotein E synthesis in human kidney, adrenal gland, and liver. Proc Natl Acad Sci USA.

[B13] Araki S, Koya D, Makiishi T (2003). APOE polymorphism and the progression of diabetic nephropathy in Japanese subjects with type 2 diabetes: results of a prospective observational follow-up study. Diabetes Care.

[B14] Araki S, Moczulski DK, Hanna L, Scott LJ, Warram JH, Krolewski AS (2000). APOE polymorphisms and the development of diabetic nephropathy in type 1 diabetes: results of case-control and family-based studies. Diabetes.

[B15] Bruschi M, Catarsi P, Candiano G (2003). Apolipoprotein E in idiopathic nephrotic syndrome and focal segmental glomerulosclerosis. Kidney Int.

[B16] Chowdhury TA, Dyer PH, Kumar S (1998). Association of apolipoprotein epsilon2 allele with diabetic nephropathy in Caucasian subjects with IDDM. Diabetes.

[B17] Eto M, Horita K, Morikawa A (1995). Increased frequency of apolipoprotein epsilon 2 allele in non-insulin dependent diabetic (NIDDM) patients with nephropathy. Clin Genet.

[B18] Feussner G, Wey S, Bommer J, Deppermann D, Grutzmacher P, Ziegler R (1992). Apolipoprotein E phenotypes and hyperlipidemia in patients under maintenance hemodialysis. Hum Genet.

[B19] Horita K, Eto M, Makino I (1994). Apolipoprotein E2, renal failure and lipid abnormalities in non-insulin-dependent diabetes mellitus. Atherosclerosis.

[B20] Kimura H, Suzuki Y, Gejyo F (1998). Apolipoprotein E4 reduces risk of diabetic nephropathy in patients with NIDDM. Am J Kidney Dis.

[B21] Roussos L, Ekstrom U, Ehle PN, Oqvist B, Floren CH (2004). Apolipoprotein E polymorphism in 385 patients on renal replacement therapy in Sweden. Scand J Urol Nephrol.

[B22] Werle E, Fiehn W, Hasslacher C (1998). Apolipoprotein E polymorphism and renal function in German type 1 and type 2 diabetic patients. Diabetes Care.

[B23] Liberopoulos E, Siamopoulos K, Elisaf M (2004). Apolipoprotein E and renal disease. Am J Kidney Dis.

[B24] Chen G, Paka L, Kako Y, Singhal P, Duan W, Pillarisetti S (2001). A protective role for kidney apolipoprotein E. Regulation of mesangial cell proliferation and matrix expansion. J Biol Chem.

[B25] Zeleny M, Swertfeger DK, Weisgraber KH, Hui DY (2002). Distinct apolipoprotein E isoform preference for inhibition of smooth muscle cell migration and proliferation. Biochemistry.

[B26] Utermann G, Langenbeck U, Beisiegel U, Weber W (1980). Genetics of the apolipoprotein E system in man. Am J Hum Genet.

[B27] Zannis VI, Just PW, Breslow JL (1981). Human apolipoprotein E isoprotein subclasses are genetically determined. Am J Hum Genet.

[B28] Hsu CC, Kao WH, Coresh J (2005). Apolipoprotein E and progression of chronic kidney disease. Jama.

[B29] Corder EH, Saunders AM, Strittmatter WJ (1993). Gene dose of apolipoprotein E type 4 allele and the risk of Alzheimer's disease in late onset families. Science.

[B30] Tang MX, Stern Y, Marder K (1998). The APOE-epsilon4 allele and the risk of Alzheimer disease among African Americans, whites, and Hispanics. Jama.

[B31] Wilson PW, Myers RH, Larson MG, Ordovas JM, Wolf PA, Schaefer EJ (1994). Apolipoprotein E alleles, dyslipidemia, and coronary heart disease. The Framingham Offspring Study. Jama.

[B32] Liberopoulos EN, Miltiadous GA, Cariolou M, Kalaitzidis R, Siamopoulos KC, Elisaf MS (2004). Influence of apolipoprotein E polymorphisms on serum creatinine levels and predicted glomerular filtration rate in healthy subjects. Nephrol Dial Transplant.

[B33] Rebeck GW, Kindy M, LaDu MJ (2002). Apolipoprotein E and Alzheimer's disease: the protective effects of ApoE2 and E3. J Alzheimers Dis.

[B34] Corder EH, Saunders AM, Risch NJ (1994). Protective effect of apolipoprotein E type 2 allele for late onset Alzheimer disease. Nat Genet.

[B35] Mahley RW, Weisgraber KH, Innerarity TL, Rall SC (1991). Genetic defects in lipoprotein metabolism. Elevation of atherogenic lipoproteins caused by impaired catabolism. Jama.

[B36] (1994). Plan and Operation of the Third National Health and Nutrition Examination Survey, 1988-1994. Series 1: Programs and Collections Procedures. Vital Health Stat.

[B37] Ezzati TM, Massey JT, Waksberg J, Chu A, Maurer KR (1992). Sample design: Third National Health and Nutrition Examination Survey. Vital Health Stat.

[B38] Crawford DC, Sanders CL, Qin X (2006). Genetic variation is associated with C-reactive protein levels in the Third National Health and Nutrition Examination Survey. Circulation.

[B39] Lohr S (1999). Sampling: Design and Analysis.

[B40] NCHS Note for Correction of Serum Creatinine for NHANES III, NHANES 1999-2000, 2001-2002 and 2003-2004. http://www.cdc.gov/nchs/data/nhanes/nhanes_03_04/general_%20note_for_serum_creatinine.pdf.

[B41] Levey AS, Coresh J, Greene T (2006). Using standardized serum creatinine values in the modification of diet in renal disease study equation for estimating glomerular filtration rate. Ann Intern Med.

[B42] Levey AS, Stevens LA, Schmid CH (2009). A new equation to estimate glomerular filtration rate. Ann Intern Med.

[B43] Astor BC, Arnett DK, Brown A, Coresh J (2004). Association of kidney function and hemoglobin with left ventricular morphology among African Americans: the Atherosclerosis Risk in Communities (ARIC) study. Am J Kidney Dis.

[B44] Gunter E, Lewis B, Kocikowski S (1996). Laboratory Procedures Used for the Third National Health and Nutrition Examination Survey (NHANES III), 1998-1994. Atlanta, GA, Public Health Service Centers for Disease Control and Prevention, National Center for Environmental Health; Hyattsville, MD, National Center for Health Statistics.

[B45] Centers for Disease Control and Prevention (CDC) National Center for Health Statistics (NCHS). National Health and Nutrition Examination Survey Laboratory Protocol Hyattsville MUSDo.

[B46] StataCorp (2005). Stata Statistical Software: Release 9.

[B47] Gauderman W, Morrison J QUANTO 1.1: A computer program for power and sample size calculations for genetic-epidemiology studies. http://hydra.usc.edu/gxe.

[B48] Garcia-Closas M, Lubin JH (1999). Power and sample size calculations in case-control studies of gene-environment interactions: comments on different approaches. American journal of epidemiology.

[B49] Levey A, Stevens L, Schmid C (2008). A New Formula to Estimate GFR from Serum Creatinine: Improved Accuracy and Updated Estimates of Prevalence of Chronic Kidney Disease in the United States.

[B50] Hsueh WC, Mitchell BD, Hixson JE, Rainwater DL (2000). Effects of the ApoE polymorphism on plasma lipoproteins in Mexican Americans. Ann Epidemiol.

[B51] Shriver MD, Boerwinkle E, Hewett-Emmett D, Hanis CL (1991). Frequency and effects of apolipoprotein E polymorphism in Mexican-American NIDDM subjects. Diabetes.

[B52] Haffner SM, Stern MP, Miettinen H, Robbins D, Howard BV (1996). Apolipoprotein E polymorphism and LDL size in a biethnic population. Arterioscler Thromb Vasc Biol.

[B53] Oda H, Yorioka N, Ueda C, Kushihata S, Yamakido M (1999). Apolipoprotein E polymorphism and renal disease. Kidney Int Suppl.

[B54] Eto M, Saito M, Okada M (2002). Apolipoprotein E genetic polymorphism, remnant lipoproteins, and nephropathy in type 2 diabetic patients. Am J Kidney Dis.

[B55] Hadjadj S, Gallois Y, Simard G (2000). Lack of relationship in long-term type 1 diabetic patients between diabetic nephropathy and polymorphisms in apolipoprotein epsilon, lipoprotein lipase and cholesteryl ester transfer protein. Genetique de la Nephropathie Diabetique Study Group. Donnees Epidemiologiques sur le Syndrome d'Insulino-Resistance Study Group. Nephrol Dial Transplant.

[B56] Greenburg A (2001). Primer on Kidney Diseases.

