# Supplementary material for: Association of APOE polymorphism with chronic kidney disease in a nationally representative sample: a Third National Health and Nutrition Examination Survey (NHANES III) Genetic Study
Source: BMC Med Genet. 2009 Oct 23;10:108. doi: 10.1186/1471-2350-10-108 (PMC2770999; doi:10.1186/1471-2350-10-108)
Supplement: Additional file 1 — Estimation of GFR using the CKD-EPI formula. The CKD-EPI formulas are provided. These formulas were used to estimate GFR in our analyses in addition to the MDRD equation. [file 1471-2350-10-108-S1.DOC]

**Additional file 1.** Estimation of GFR using the CKD-EPI formula.

| **CKD-EPI formula** | |  | |
| --- | --- | --- | --- |
| **Race** | **Sex** | ***SCr* (mg/dl)** | **GFR formula** |
| Black | Female | ≤0.7 | 161 x (0.993)^Age^  x (*SCr*/0.7)^-0.329^ |
| Black | Female | >0.7 | 161 x (0.993)^Age^ x (*SCr*/0.7)^-1.209^ |
| Black | Male | ≤0.9 | 163 x (0.993)^Age^ x (*SCr*/0.9)^-0.411^ |
| Black | Male | >0.9 | 163 x (0.993)^Age^ x (*SCr*/0.9)^-1.209^ |
| White or other | Female | ≤0.7 | 139 x (0.993)^Age^ x (*SCr*/0.7)^-0.329^ |
| White or other | Female | >0.7 | 139 x (0.993)^Age^ x (*SCr*/0.7)^-1.209^ |
| White or other | Male | ≤0.9 | 141 x (0.993) ^Age^ x (*SCr*/0.9)^-0.411^ |
| White or other | Male | >0.9 | 141 x (0.993)^Age^ x (*SCr*/0.9)^-1.209^ |
